# Reporting of key methodological and ethical aspects of cluster trials in hemodialysis require improvement: a systematic review

**DOI:** 10.1186/s13063-020-04657-9

**Published:** 2020-08-28

**Authors:** Ahmed A. Al-Jaishi, Kelly Carroll, Cory E. Goldstein, Stephanie N. Dixon, Amit X. Garg, Stuart G. Nicholls, Jeremy M. Grimshaw, Charles Weijer, Jamie Brehaut, Lehana Thabane, P. J. Devereaux, Monica Taljaard

**Affiliations:** 1 Lawson Health Research Institute, London, ON Canada; 2 Department of Health Research Methods, Evidence, and Impact, McMaster University, Hamilton, ON Canada; 3 ICES, Toronto, Canada; 4 Clinical Epidemiology Program, Ottawa Hospital Research Institute, Ottawa, ON Canada; 5 Department of Philosophy, Western University, London, ON Canada; 6 Department Medicine, Epidemiology and Biostatistics, Western University, London, ON Canada; 7 Department of Mathematics and Statistics, University of Guelph, Guelph, ON Canada; 8 Department of Medicine, University of Ottawa, Ottawa, ON Canada; 9 School of Epidemiology and Public Health, University of Ottawa, Ottawa, ON Canada

**Keywords:** Cluster randomized controlled trial, Systematic review, Ethics, Informed consent, Hemodialysis

## Abstract

**Background:**

The hemodialysis setting is suitable for trials that use cluster randomization, where intact groups of individuals are randomized. However, cluster randomized trials (CRTs) are complicated in their design, analysis, and reporting and can pose ethical challenges. We reviewed CRTs in the hemodialysis setting with respect to reporting of key methodological and ethical issues.

**Methods:**

We conducted a systematic review of CRTs in the hemodialysis setting, published in English, between 2000 and 2019, and indexed in MEDLINE or Embase. Two reviewers extracted data, and study results were summarized using descriptive statistics.

**Results:**

We identified 26 completed CRTs and five study protocols of CRTs. These studies randomized hemodialysis centers (*n* = 17, 55%), hemodialysis shifts (*n* = 12, 39%), healthcare providers (*n* = 1, 3%), and nephrology units (*n* = 1, 3%). Trials included a median of 28 clusters with a median cluster size of 20 patients. Justification for using a clustered design was provided by 15 trials (48%). Methods that accounted for clustering were used during sample size calculation in 14 (45%), during analyses in 22 (71%), and during both sample size calculation and analyses in 13 trials (42%). Among all CRTs, 26 (84%) reported receiving research ethics committee approval; patient consent was reported in 22 trials: 10 (32%) reported the method of consent for trial participation and 12 (39%) reported no details about how consent was obtained or its purpose. Four trials (13%) reported receiving waivers of consent, and the remaining 5 (16%) provided no or unclear information about the consent process.

**Conclusion:**

There is an opportunity to improve the conduct and reporting of essential methodological and ethical issues in future CRTs in hemodialysis.

**Review Registration:**

We conducted this systematic review using a pre-specified protocol that was not registered.

## Introduction

Patients on hemodialysis are often excluded from clinical trials, and many trials in the hemodialysis setting suffer from poor recruitment, inadequate sample sizes, and poor adherence to allocated treatment and treatment contamination [1–5]. Cluster randomized trials (CRTs) randomize intact groups of individuals (rather than independent individuals) to different arms. This design can offer a logistically convenient method to produce high-quality evidence, can be effective in avoiding treatment contamination, and may be better received by participants and healthcare staff when delivered to a group of individuals rather than select patients. The CRT is an attractive design in the hemodialysis setting, where interventions are often delivered at the center-level and where staff follow the same protocol for patients under their care.

Cluster randomization, however, introduces methodological issues that need to be addressed during the design and analysis stages [6, 7]. First, it may not be possible to identify and recruit participants until after the cluster has been randomized. This increases the risk of selection bias because knowledge of the allocated arm can influence both the identification of potential participants and their decisions to participate. Second, because outcomes are usually correlated within clusters, CRTs are statistically less efficient than individual-level randomized trials. As such, the CONSORT Statement for Cluster Randomized Trials requires that studies report how clustering was considered in both sample size calculation and analysis. Failing to account for clustering in the sample size calculation implies that the study may not have adequate power to detect meaningful differences between the groups, while failing to account for clustering in the analysis implies that standard errors of treatment effects will be under-estimated, increasing the risk of spurious statistical significance [6–9].

The CRT design also raises complex ethical issues. *The Ottawa Statement on the Ethical Design and Conduct of Cluster Randomized Trials* offers ethical guidance, providing 15 recommendations for those who design, conduct, and review CRTs [10–14]. For example, ethical issues that may challenge researchers including the following: When is a study considered research? Who is the research subject? And from whom, how, and when must informed consent be obtained? A summary of the Ottawa Statement recommendations and applicability of these recommendation for CRTs conducted in the hemodialysis setting is provided in Additional file 1: Appendix 1.

In the present study, we conducted a descriptive analysis of how CRTs in hemodialysis report key methodological (with regards to accounting for clustering effects and reporting of intra-class correlation coefficient) and ethical issues (with regards to the elements highlighted in the Ottawa Statement). This review will serve as a foundational step in a multi-year initiative that seeks to develop recommendations for the ethical design, conduct, and reporting of CRTs in the hemodialysis setting.

## Materials and methods

### Protocol and registration

We conducted this systematic review using a pre-specified protocol and reported our results according to published guidelines (PRISMA Checklist: Additional file 1: Appendix 2) [15].

### Studies eligible for review

We did not set any limits on country of study and included published primary reports of CRTs or study protocols of CRTs with an unpublished primary report. We aimed to include English-language reports published between January 2000 and November 2019 that involved (1) patients on in-center hemodialysis or( 2) patients on in-center hemodialysis as a subgroup in a larger study of non-in-center hemodialysis patients. When we found a study protocol of a CRT with an identified completed trial, we used the protocol to supplement any missing information from the final published report. Other reports such as secondary analyses, conference abstracts, and pilot or feasibility CRTs were excluded. We excluded feasibility and pilot trials because they have different methodological [16] and ethical considerations than full scale CRTs.

### Information sources

We implemented a search syntax on November 30, 2019, to identify published reports in MEDLINE and Embase.

### Search

Our search strategy combined two published search filters designed to identify publications related to CRT [17] and dialysis [18] studies (Additional file 1: Appendix 3). Two reviewers (AAA and KC) screened titles and abstracts of articles. AAA manually searched for additional articles in bibliographies of all included articles, list of articles that cited the included studies in Google Scholar, and “Similar articles” feature in PubMed. The complete list of included studies was also reviewed by an expert in the field (AXG) to capture additional studies that may have been missed.

### Study selection

We retrieved the full text of any article considered potentially relevant by any reviewer. Full-text articles were assessed for study eligibility by two reviewers (AAA and KC), with disagreements resolved through discussion. Agreement between the two reviewers was evaluated using the Kappa statistic [19].

### Data collection process

We utilized a data abstraction form that was pilot tested on three studies by three reviewers (AAA, KC, and CEG). Thereafter, two reviewers (AAA and either KC or CEG) independently extracted data from each manuscript. Extracted details on trials considering the effect of clustering during sample size estimation and analysis were completed by AAA and either MT or SND. After each set of three studies, data extractions were compared within the pair and disagreements resolved by consensus. Details of extracted data are highlighted in Additional file 1: Appendix 4. We extracted data on study characteristics, methodological characteristics, data collection method, justification for using a CRT design, type of intervention, information regarding research ethics committee review, gatekeepers (i.e., an individual or body that represents the interests of cluster members, clusters, or organizations [20]), informed consent procedures, and any information about harm-benefit assessment or protection of vulnerable populations. Kidney disease disproportionally affects individuals traditionally considered vulnerable (e.g., patients with dementia). We defined vulnerable participants as any research participants who “may have increased likelihood of being wronged or of incurring additional harm,” as per the CIOMS international ethics guidelines [21]. This includes persons who have “impairments in decisional capacity, education, resources, strength, or other attributes needed to protect their own interests [21]”. We coded the presence of any vulnerable participants as clearly present, potentially present or unclear, and clearly absent or not relevant. If a vulnerability was clearly or potentially present, we looked for any reporting of additional protections provided by the authors.

### Analysis

We summarized results using frequencies for categorical variables and medians with interquartile ranges for continuous variables. Given the small number of included studies, we did not test changes in reporting over time nor association between reporting of ethical elements and study characteristics. For all our analyses, we used R (Version 3.6.2) [22].

## Results

### Characteristics of included studies

**Fig. 1:**
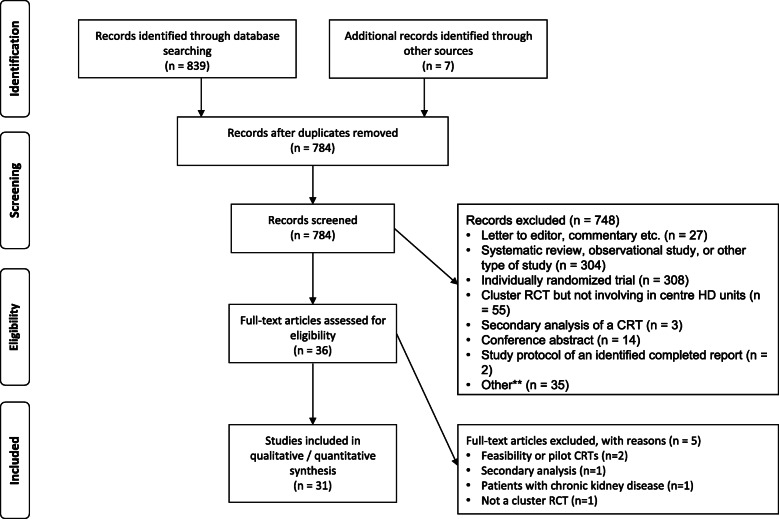
Flow diagram of study selection. **Other: One manuscript described the statistical plan for a main publication not related to cluster randomized trials, two described a program of research not related to the target population, and two were duplicate records not previously removed. Abbreviation: RCT, randomized controlled trial; CRT, cluster randomized trial

The study flow diagram is presented in Fig. 1. We screened 777 citations and retrieved 29 full-text articles to assess eligibility. We identified another seven articles by reviewing citation links (*n* total = 36). We had almost perfect between-reviewer agreement on which studies met the criteria for review (kappa statistic 0.96, 95% confidence interval: 0.91 to 1.00). Five articles were excluded after full-text review [23–27]. Thus, 31 articles were included in this review: 26 completed studies and 5 study protocols [28–58].

Study characteristics for the included trials are presented in Table 1. The 31 trials were published in 19 journals. Nineteen trials (61%) recruited patients from the USA, three (10%) were from the UK, three (10%) from Australia/New Zealand, and seven (23%) from other countries (some trials were multi-national and these categories are not mutually exclusive).

**Table 1:** Included studies and their characteristics

First author	Year	Country	Intervention arm (number)		Control arm (number)		Type of cluster	Type of patients^**₳**^	Type of intervention^**₱**^	Primary outcome
			Clusters	Patients	Clusters	Patients				
Sehgal [28]	2002	USA	21	85	23	84	Individual providers	Prevalent only	2 and 3	Change in Kt/V and achievement of facility Kt/V goal
McClellan [29]	2004	USA	21	2237	20	2044	HD units	Prevalent and incident	1, 2, and 3	Proportion of patients whose urea reduction ratio was ≥65%
Leon [40]	2006	USA	21	86	23	94	HD units	Prevalent only	3	Serum albumin level
Pradel [51]	2008	USA	14	107	14	107	Shifts in HD unit	Prevalent and incident	3	See ^¥^
Locatelli [53]	2009	EU**	NR*	321	NR	278	Nephrology Unit	Prevalent and incident	2	Proportions of patients with hemoglobin > 11 g/dL, serum ferritin > 100 μg/L, hypochromic red cell count < 10%, or transferrin saturation > 20%
Sullivan [48]	2009	USA	14	145	14	134	Shifts in HD unit	Prevalent only	3	Serum phosphorus level
Bond [49]	2011	USA	38	3157	39	3135	HD units	Prevalent and incident	2 and 3	Change in influenza vaccination rates
Kauric-Klein [54]	2012	USA	NR	59	NR	59	HD Units	Prevalent only	3	Changes in systolic blood pressure over time (primary outcome not explicitly stated)
Sullivan [50]	2012	USA	11	92	12	75	HD units	Prevalent and incident	3	Number of transplant process steps completed
Bennett [55]	2013	AUS/NZ	2	38	2	41	HD units	Prevalent and incident	2	Rate of referral to dietetic services for nutrition support
Karavetian [56]	2013	Lebanon	1	37	1	24	Shifts in HD unit	Prevalent and incident	3	Patient knowledge score^£^
Weisbord [57]	2013	USA	9	100	9	120	Shifts in HD unit	Prevalent and incident	2	Changes in scores on pain, erectile dysfunction and depression surveys
Rosenblum [58]	2014	USA	216	4609	216	4551	HD units	Prevalent and incident	2 and 4	Positive blood culture rate
Wileman [30]	2014	UK	6	45	6	45	Shifts in HD unit	Prevalent and incident	3	Serum phosphate level
Karavetian [31]	2015	Lebanon	6	88	6	96	Shifts in HD unit	Prevalent and incident	3	Serum phosphorus level
Bennett [32]	2016	AUS/NZ	15	171	15	171	HD units	Prevalent and incident	3 and 4	30-s sit-to-stand test
Graham-Brown [33]	2016	UK	3	NA***	3	NA***	Shifts in HD unit	Prevalent only	4	Left ventricular mass
Howren [34]	2016	USA	11	61	11	58	Shifts in HD unit	Prevalent and incident	3	Unclear: Mean interdialytic weight gain across for periods **or** Fluid nonadherent as defined by an interdialytic weight gain > 2.5 kg over a 4-week period
Wileman [35]	2016	UK	6	49	6	40	Shifts in HD unit	Prevalent and incident	3	Interdialytic weight gain
Hymes [36]	2017	USA	20	1245	20	1225	HD units	Prevalent and incident	2 and 4	Positive blood culture rate
Patzer [37]	2017	USA	67	4203	20	1225	HD units	Prevalent and incident	1, 2, and 3	Facility level transplant referral rate
Patzer [38]	2017	USA	NA***	NA***	NA***	NA***	HD units	Prevalent and incident	1, 2, and 3	Co-primary outcomes of (i) change in proportion of patients waitlisted and (ii) disparity reduction in proportion of patients waitlisted in a dialysis facility after 1 year
Brunelli [39]	2018	USA	20	826	20	845	HD units	Prevalent and incident	4	Positive blood culture rate
Delmas [41]	2018	Switzerland	NR	NR	NR	NR	HD Units	Prevalent only	1	Nurse quality of working life
Griva [42]	2018	Singapore	14	101	14	134	Shifts in HD unit	Prevalent only	3	Serum potassium/phosphate levels and interdialytic weight gains
Huang [43]	2018	China	1	46	1	44	Shifts in HD unit	Prevalent and incident	3	Blood pressure monitored before each hemodialysis
Milazi [44]	2018	AUS/NZ	3	60	3	60	Shifts in HD unit	Prevalent and incident	3	Serum phosphate level
Song [52]	2018	USA	NA***	NA***	NA***	NA***	HD units	Prevalent only	3 and 5	Patient and surrogate self-reported preparedness for end-of-life decision making
Sullivan [45]	2018	USA	20	1041	20	836	HD units	Prevalent and incident	3	Placement on kidney transplant waiting list
Waterman [46]	2018	USA	10	133	10	120	HD units	Prevalent and incident	3	Patients’ readiness to allow someone to be a living donor
Dember [47]	2019	USA	133	1938	133	2532	HD units	Incident only	4	Death

*NR* not reported, *USA* United States of America, *EU* European Union, *UK* United Kingdom, *AUS/NZ* Australia/New Zealand, *NA* not applicable, *g/dL* grams per deciliter, *μg/L* micrograms per liter, *Kt/V* fractional urea clearance represented by *K* = dialyzer clearance of urea, *t* = dialysis time, *V* = distribution volume of urea

*Locatelli et al. did not report the number of clusters randomized to each arm; however, the authors reported a total of 53 nephrology units participated in the trial

**Included countries from Bulgaria, Croatia, Poland, Romania, and Serbia and Montenegro

***This was a study protocol of an ongoing trial and thus the final sample size used (or to be used) in the analysis was not available

^₳^We defined patients as “prevalent” if they were on hemodialysis for at least 6 months and “incident” if they are newly starting or started hemodialysis less than 6 months prior to baseline

^¥^Study assessed three distinct behaviors to explore patients’ readiness to pursue living donor kidney transplant: (1) considering living donor kidney transplant, (2) talking with family or friends about living donor kidney transplant, and (3) asking someone to be a kidney transplant donor

^£^Patient knowledge questionnaire was utilized to assess patients’ knowledge of kidney disease, renal diet, phosphate binders, and vitamin D therapy

^₱^1 = educational/ quality improvement interventions targeted at health professionals (e.g., transplant education and engagement activities targeting health professionals, etc.); 2 = quality improvement interventions targeted at organization of health care or health services delivery (e.g., nutrition screening, change in catheter exit-site care, etc.); 3 = patient health promotion or educational intervention (e.g., education about benefits of resistance exercise program, dietary counseling, education on avoiding foods with phosphorus additives, etc.); 4 = direct patient therapeutic intervention (e.g., intradialytic resistance training, antimicrobial barrier caps for catheters, etc.); and 5 = other

### Reporting of methodological characteristics

Table 2 provides a description of the reporting of study characteristics. Thirty trials (97%) utilized a parallel arm design and one trial (3%) used a stepped-wedge design. All trials were designed as superiority trials. The types of randomized clusters were hemodialysis centers (*n* = 17; 55%), hemodialysis shifts or sessions (*n* = 12; 39%), providers or professionals (*n* = 1; 3%), and nephrology units (*n* = 1; 3%; it was not clear how a “nephrology unit” was defined). Clusters were randomly allocated to the treatment arm using unrestricted (*n* = 8; 26%), pair-matched (*n* = 4; 13%), stratified (*n* = 4; 13%), split-cluster (*n* = 11, 35% [i.e., day shifts within centers]), covariate-constrained randomization (*n* = 1, 3%), or an unreported method of allocation (*n* = 3, 10%).

**Table 2:** Reporting of study characteristics

Component	Number of studies (%) (***N*** total = 31)
**Trial design**	
Parallel arm	30 (97%)
Stepped-wedge design	1 (3%)
**Types of randomized clusters**	
Hemodialysis centers	17 (55%)
Hemodialysis shifts or sessions	12 (39%)
Providers or professionals	1 (3%)
Nephrology units^₱^	1 (3%)
**Method of random allocation**	
Completely randomized design (unrestricted randomization)	8 (26%)
Stratified design	4 (13%)
Pair-matched design	4 (13%)
Split-cluster (i.e., shifts within a hemodialysis center)	11 (35%)
Covariate-constrained	1 (3%)
Not reported	3 (10%)
**Number of clusters per trial [median (25th, 75th percentile)]¥**	28 (12, 43)
**Number of patients per trial [median (25th, 75th percentile)]**^**₳**^	228 (120, 1723)
**Number of patients per cluster [median (25th, 75th percentile)]**^**€,Ϫ**^	20 (8, 32)

^₱^It is not clear how a “nephrology unit” was defined

Estimate is based on ^¥^32, ^₳^29, and ^€^28 trials. Missing data may have been a result of not reporting or the study being a protocol with no final information on the number of clusters/patients being available

^Ϫ^For each study, we estimated the average cluster size by dividing the total number of patients recruited by the number of clusters (e.g., 200 patients recruited in a trial/10 clusters = 20 patients per cluster). We then took the median of the calculated average of patients per clusters from each trial

The median (25th, 75th percentile) number of clusters included per trial was 28 (12, 43), and all trials used 1:1 randomization. One trial (3%) had one cluster per arm, and six trials (19%) had fewer than the minimum recommendation of four clusters per arm [7, 8]. The median number of participants per trial was 228 (120, 1723). All trials included patients (as opposed to providers alone) as the research participants with a median number of 20 (8, 32) patients per cluster.

One study (3%) reported the intra-class correlation coefficient (ICC) for their primary outcome. Table 3 describes whether and how clustering was accounted for during sample size estimation and analysis. Fourteen trials (45%) accounted for clustering during sample size estimation for the primary outcome, three (10%) did not account for clustering, two (6%) accounted for clustering but using a different outcome measure than the primary outcome, one (3%) was unclear, and 11 (35%) did not report a sample size or power estimate. At the analysis stage, 22 trials (71%) accounted for clustering using either an individual-level analysis adjusting for clustering or using a cluster-level summary method. The remaining nine trials (29%) either did not account for clustering in their primary analysis or it was unclear if clustering was accounted for in the analysis. A total of 13 trials (42%) accounted for clustering in both the sample size calculation and analysis.

**Table 3:** Reporting of (a) how clustering was considered during sample size estimation and analysis and (b) justification for using a cluster randomized design

	***N*** = 31 trials (%)
**Did sample size/power calculations account for the cluster design?**	
Not presented^₳^	11 (35%)
Yes, used patient-level data and accounted for clustering (e.g., random effects model)	11 (35%)
Yes, used cluster-level summaries	3 (10%)
No, used patient-level data without accounting for clustering	3 (10%)
Unclear	1 (3%)
Other^¥^	2 (6%)
**Did the analysis for primary outcome account for clustering?**	
Yes, used patient-level data and accounted for clustering	17 (55%)
Yes, used cluster-level summaries	5 (16%)
No, used patient-level data without accounting for clustering ₱	7 (23%)
Unclear/other^¥^	2 (6%)
**Justification for utilizing a cluster randomized design** (categories were not mutually exclusive)	
None provided	16 (52%)
Avoid contamination	15 (48%)
Logistical or administrative convenience	2 (6%)

^₳^One study presented power calculation, but it was a post hoc power analysis

^¥^This may have included using an inappropriate method for the proposed primary outcome, or the study accounted for clustering but not based on the primary outcome measure (e.g., they assumed a continuous outcome, but the primary endpoint was a proportion)

^₱^One study accounted for repeated events within patients but did not report accounting for within-cluster correlation; another study reported using a generalized linear mixed model but did not specify whether they accounted for the effect of the cluster as random effect

### Reporting of justification for cluster randomization

Of all 31 trials, 15 trials (48%) reported a justification for using a cluster randomized design (Table 3). Thirteen trials (42%) reported using a CRT design to avoid contamination and two trials (6%) reported using a CRT design to avoid contamination and for logistical/administrative convenience.

### Reporting of intervention type and target population

Table 4 lists the types of intervention used in each arm of included trials. The most common type of study intervention was health promotion or an educational intervention (*n* = 22 trials; 71%) for which patients were the intended recipients. Six trials (19%) examined a direct patient therapeutic intervention—for example, intradialytic resistance training or antimicrobial barrier caps for central venous catheters. Among all trials, the intervention was necessarily administered at the cluster-level (e.g., education of providers) for 18 trials (58%). In the control arm, 23 trials (74%) utilized “usual care,” four (13%) used some form of augmented care (usual care plus some minimal elements of active intervention), three (10%) used an active control, and one (3%) used an attention-placebo. Four trials (13%) utilized interventions that included an educational or quality improvement component targeting health professionals (e.g., transplant education and engagement activities). Both prevalent and incident patients on hemodialysis were included in 22 trials (71%), eight trials (26%) included only prevalent patients, and one trial (3%) included only incident patients on hemodialysis.

**Table 4:** Summary of results for type(s) of interventions, data collection procedures, reporting of participant consent procedures for study interventions and data collection, timing of any participant consent, and whether participants can opt out of the intervention or data collection

Component	Intervention arm ***n*** (%)	Control arm ***n*** (%)
**Type(s) of interventions (i.e., all components of intervention)¥**	***N*** **total = 31**	***N*** total = 8**
Educational/ quality improvement interventions targeted at health professionals (e.g., transplant education and engagement activities targeting health professionals, etc.)	4 (13%)	0 (0%)
Quality improvement interventions targeted at organization of health care or health services delivery (e.g., nutrition screening, change in catheter exit-site care, etc.)	10 (32%)	2 (25%)
Patient health promotion or educational intervention (e.g., education about benefits of resistance exercise program, dietary counseling, education on avoiding foods with phosphorus additives, etc.)	22 (71%)	4 (50%)
Direct patient therapeutic intervention (e.g., intradialytic resistance training, antimicrobial barrier caps for catheters, etc.)	6 (19%)	1 (12%)
Other €	1 (3%)	1 (12%)
**Types of Data collection procedures ¥**	***N*** **total = 31**	***N*** **total = 31**
Routinely collected outcomes extracted locally from existing patient medical records (physical charts or electronic records)	30 (97%)	30 (97%)
Data query from clinical data registry or other central source of routinely collected data (e.g., administrative data)	11 (35%)	11 (35%)
Specimen collection or physical examination that were not required for usual patient care	4 (13%)	4 (13%)
Interviewer-administered patient questionnaires done face-to-face or by telephone that were not required for usual patient care	9 (29%)	9 (29%)
Self-administered patient questionnaires (done by mail, e-mail or Internet) that were not required for usual patient care	18 (58%)	16 (52%)
Other ₳	5 (16%)	2 (6%)
**Reporting of participant consent procedures for** ***study interventions***	***N*** **total = 31**	***N*** **total = 31**
Reported written informed consent	9 (29%)	10 (32%)
Reported verbal informed consent	1 (3%)	0 (0%)
Reported informed consent but no details about method or what consent was for	12 (39%)	11 (35%)
Reported the study was exempt from research ethics committee review, received waiver of consent, or explicitly stated no consent	4 (13%)	4 (13%)
Unclear if participants consented	1 (3%)	2 (6%)
Not mentioned	4 (13%)	4 (13%)
**Reporting of participant consent procedures for** ***data collection***	***N*** **total = 31**	***N*** **total = 31**
Reported written informed consent	7 (22%)	6 (19%)
Reported verbal informed consent	1 (3%)	1 (3%)
Reported informed consent but no details about method or what consent was for	14 (45%)	14 (45%)
Reported the study was exempt from research ethics committee review, received waiver of consent, or explicitly stated no consent	4 (13%)	4 (13%)
Unclear if participants consented	1 (3%)	2 (6%)
Not mentioned	4 (13%)	4 (13%)

^¥^The responses to these questions were not mutually exclusive

^₳^Active data collection, including using case report form

^€^Surrogate decision-maker educational intervention in the intervention arm; audit feedback from previous year in the control arm

**These questions were not applicable when the comparator arm was ***usual care***

### Data collection procedures

Data collection procedures in the intervention and control arm were similar for most trials (Table 4). In the intervention arm, 30 (97%) trials used local routinely collected data (e.g., medical charts or electronic medical records) as the primary source for data collection. Eleven trials (35%) used clinical registry data and 24 (77%) supplemented routinely collected data with additional sources: self-administered questionnaires (*n* = 18; 58%), interviewer administered questionnaires (*n* = 9; 29%), specimen collection or physical examination not required for usual patient care (*n* = 4; 13%), as well as active data collection (*n* = 5; 16%), for example, using case report forms.

### Gatekeepers

Five trials (16%) reported that a gatekeeper provided permission for clusters to participate in the study (Table 5). For the remaining trials (84%), no information about gatekeepers was provided.

**Table 5:** Summary of results for reported information about gatekeepers, research ethic committee review, timing of any participant consent, and whether participants can opt out of the intervention or data collection

Component	Number of trials ***N*** total = 31 (%)
**Whether a gatekeeper was identified that allowed access to each cluster**	
Yes—a clearly identified individual or body	3 (10%)
Yes—but the gatekeeper not clearly identified	2 (6%)
No gatekeeper information provided	26 (84%)
**Reporting of research ethics review**	
Stated research ethics committee approval	26 (84%)
Stated research ethics committee exempt (specify reason)	1 (3%)
Not reported	4 (13%)
**Timing of any participant consent**	
Not applicable	4 (13%)
Any consent was ***before*** randomization of clusters	7 (23%)
Any consent was ***after*** randomization of clusters	10 (32%)
Timing of consent was unclear and could not be deduced from the report	10 (32%)
**Whether participants can opt out of the data collection**	
Yes—it is clearly reported that participants could opt out of data collection	7 (23%)
No—participants could not opt out of data collection	3 (10%)
Not reported or Unclear if participants could opt out	21 (68%)
Presence of any vulnerable participants* in the trial	
Clearly present	6 (20%)
Potentially present or unclear	24 (77%)
Clearly absent or not relevant	1 (3%)

### Reporting of research ethics review

We found that 26 trials (84%) reported research ethics committee approval, one (3%) reported that the study was exempt from review, and four (13%) did not report whether the study was reviewed by a research ethics committee (Table 5).

### Reporting of consent procedures

One trial (3%) reported they received an exemption from ethics review, three (10%) received a waiver of consent from the research ethics committee (see Additional file 1: Appendix 5), 22 trials (71%) reported obtaining consent from patients, and five (16%) trials either did not discuss the consent process or it was unclear if patients provided informed consent. For the 22 trials (71%) that reported obtaining consent from patients, written or verbal informed consent was reported in 10 trials (45%) for the study intervention and eight (36%) for data collection; the remaining trials provided no details about the method of consent for study intervention and/or for data collection (Table 4).

Among the remaining 27 trials that did not receive an exemption from ethics review or had a waiver of consent, the timing of consent took place before randomization for seven trials (26%), after randomization for 10 (37%), and was unclear for the remaining 10 trials (37%).

The ability for participants to opt out of the data collection was reported in seven trials (23%); three trials (10%) reported patients could not opt out of data collection, and ability to opt out was unclear for the remaining 21 trials (68%) (Table 5).

### Assessment of benefit-harm and protections for vulnerable groups

Vulnerable participants were clearly present in 6 trials (20%); in another 24 (77%), vulnerable participants were considered to be potentially present or their presence could not be clearly ruled out (Table 5). None of the trials reported additional protections for vulnerable patients.

## Discussion

The hemodialysis population is suitable for the CRT design, especially for interventions that are implemented at the center-level; in our review, approximately 60% of trials utilized an intervention that was necessarily administered at the cluster-level. This review presents a descriptive analysis of the reporting of key methodological and ethical characteristics of CRTs involving hemodialysis patients. Guidance on the reporting of CRTs is provided in the CONSORT extension for CRTs, while the Ottawa Statement is currently the only guidance document specific to the ethical design and conduct of CRTs in health research [8, 14]. While several studies were published prior to the dissemination of the CONSORT, the Ottawa Statement or both, the interpretation of our results would not change had we presented our results based on the period pre- and post-publication of these statements.

We found that cluster randomized trials in hemodialysis have poor methodological quality and sub-optimally report ethical considerations around this design. While many of the identified issues are not unique to the hemodialysis setting, we consider three issues that require special attention: (1) taking clustering into account at the sample size estimation and analysis stages, (2) methodological and contamination issues around designs that randomize shifts within hemodialysis centers, and (3) reporting on how the rights of vulnerable participants are protected.

First, patients on hemodialysis within the same center have similar characteristics compared to patients from other centers. For example, small satellite hemodialysis centers might have patients that are medically stable compared to large academic centers that might treat sicker patients requiring close medical monitoring. It is concerning that more than half of included trials did not report a method that appropriately accounts for within-cluster correlation when estimating sample size and more than a quarter of trials did not account for clustering in the analysis, putting the study results at an increased risk of spurious statistical significance [7–9, 14]. Adjusting for clustering is especially important in this setting because there is generally high practice variation between hemodialysis centers and low variation with-in centers [59–61], factors that increase the ICC [62].

Currently, there is limited information in the literature to inform estimates of the ICC for outcomes of patients on hemodialysis; thus, researchers in hemodialysis must rely on estimates from other disciplines or historic data. As such, it is important for completed trials to report the observed ICC or design effect estimates for their outcomes so that the community can begin to build a repository that might help in the design of future trials. In our review, only one trial reported an ICC [47].

Second, a common experimental design was to randomize shifts within hemodialysis centers (e.g., Mon, Wed, Fri versus Tues, Thu, Sat). This type of randomization requires additional considerations in design and conduct. For example, the same healthcare staff will care for patients dialyzing in a single center in both arms of the trial. Contamination of the two arms of the trial can still occur if staff observe better patient outcomes in one arm and then begin to implement the treatment in clusters (i.e., shifts) in the other arm. This type of design also requires additional considerations in the analysis because clustering can occur at two levels, i.e., center and shift. It was beyond this review to assess whether authors reported the appropriate analyses accounting for this type of experimental design.

Third, authors should report how the rights of vulnerable participants are protected, especially those that have limited health literacy or may not be capable to provide informed consent. When including these subgroups, it raises ethical concerns about the extent to which these participants are truly informed. There are no clear standards for “how much” understanding is adequate [63]. Additionally, lower education levels, lower health literacy, and a participant’s primary language are all associated with poor comprehension of the informed consent process [64]. These characteristics are particularly important in the hemodialysis setting, where vulnerable participants are overrepresented [65–68].

In general, trials in this setting were small with both a limited number of clusters and patients within clusters. One trial randomized only one cluster to each arm of the trial and a fifth of reviewed trials had four or fewer clusters [43, 56]. Randomizing two clusters effectively precludes any inferences about the intervention because it is impossible to disentangle natural variation between clusters from the effect of the intervention [69]. While some have suggested that parallel arm CRTs should have at least four clusters per study arm [7, 8], with such a small number of clusters, the study may be severely under-powered, parametric statistical tests (e.g., *t* tests) may not meet the assumption of normality, and there is a high risk of baseline imbalances between trial arms that might complicate the interpretation of the trial results [70].

There is room for improved reporting of consent procedures. When consent is required, study authors ought to report adequate details to assess what consent was for (e.g., enrollment, receiving the interventions, data collection), as well as from whom (e.g., patients, providers, etc.), when (before or after randomization), and how (e.g., written, verbal) consent was obtained [8, 14]. The timing of informed consent was either not reported or took place after randomization for 20 trials. Post-randomization consent, especially when the study is unblinded, is a key risk of bias that can introduce selection bias through differential recruitment [8]. When applicable, researchers must justify how their study meets the criteria for a waiver or alteration of informed consent as outlined by national regulations or international guidelines [21, 71, 72].

Our study has several limitations. We were unable to examine changes in quality of reporting over time, or factors associated with better reporting due to the small number of trials. When a study protocol was published for one of the completed trials, we used both references to complete study extraction; however, we did not have access to the original research ethics submissions or non-peer-reviewed study protocols, did not follow-up with study authors, and did not conduct a search of any trial registries or Green Open Access options (e.g., ResearchGate). Thus, our results are based exclusively on what was reported in peer-reviewed published articles; for example, we are aware of other ongoing CRTs not included here because no study protocol or a primary report was available at the time of our search [73, 74].

Our study also has several strengths. We utilized an abstraction tool that has been developed and refined over several studies [75–78]. It is unlikely that a substantial number of relevant primary trials were missed, as we combined two validated search strategies supplemented with an extensive manual search of reference resources [17, 18]. To reduce the risk of misclassification of trial characteristics and reporting practices, we used consensus between two reviewers who independently extracted information from published articles.

## Conclusion

There is suboptimal conduct and reporting of methodological issues of CRTs in the hemodialysis setting and incomplete reporting of key ethical issues. The *Ottawa Statement on the Ethical Design and Conduct of Cluster Randomized Trials* provides specific recommendations for CRTs, but did not consider unique characteristics of the hemodialysis setting [14]. This systematic review was conducted as a first step to describe key study design characteristics and document reporting of ethical practices in CRTs in the hemodialysis setting. Our future work builds on the information from this review to explore the views/perceptions of researchers and patients with regard to the ethical issues for CRTs in the hemodialysis setting.

## Supplementary information

**Additional file 1: Appendix 1.** Recommendations from the Ottawa Statement. **Appendix 2.** PRISMA Checklist. **Appendix 3.** Search syntax to identify relevant articles in Medline between January 1st, 2000 and July 20th, 2018 in Embase Classic+Embase, Ovid MEDLINE(R) Epub Ahead of Print, In-Process & Other Non-Indexed Citations, Ovid MEDLINE(R) Daily and Ovid MEDLINE(R). **Appendix 4.** Extracted data. **Appendix 5.** Reported information about waiver of consent for the four studies that reported a waiver of informed patient consent or research ethic committee exemption.

## Data Availability

Study data can be made available upon request.
