# Supplementary material for: Reporting of key methodological and ethical aspects of cluster trials in hemodialysis require improvement: a systematic review
Source: Trials. 2020 Aug 28;21:752. doi: 10.1186/s13063-020-04657-9 (PMC7456003; doi:10.1186/s13063-020-04657-9)
Supplement: Supplementary file 1 — Additional file 1: Appendix 1. Recommendations from the Ottawa Statement. Appendix 2. PRISMA Checklist. Appendix 3. Search syntax to identify relevant articles in Medline between January 1st, 2000 and July 20th, 2018 in Embase Classic+Embase, Ovid MEDLINE(R) Epub Ahead of Print, In-Process & Other Non-Indexed Citations, Ovid MEDLINE(R) Daily and Ovid MEDLINE(R). Appendix 4. Extracted data. Appendix 5. Reported information about waiver of consent for the four studies that reported a waiver of informed patient consent or research ethic committee exemption. [file 13063_2020_4657_MOESM1_ESM.docx]

**Appendix 1**: Recommendations from the Ottawa Statement

| **Ethical Issue** | **Recommendation**  **Number** | **Recommendation** | **How trials in our review reported on these recommendations** |
| --- | --- | --- | --- |
| Justification for using cluster randomization | 1 | Researchers should provide a clear rationale for the use of the cluster randomized design and adopt statistical methods appropriate for this design. | Cluster randomized trials in the hemodialysis setting often fail to provide a clear rationale for the choice of cluster randomization. Providing a clear rationale is especially important in the case of individual-level interventions, in which a patient randomized trial would have been possible. In such circumstances, researchers should explain why the benefits of cluster randomization outweigh the disadvantages of increased sample size and risks of bias.  The effects of clustering must be considered during sample size calculation and analysis to avoid an underpowered study and spurious statistical significance, respectively. Additionally, when there are multiple levels of clustering (e.g. multiple hemodialysis shifts or providers within centres), all levels of clustering may need to be taken into consideration in the design and analysis. |
| Research ethics committee review | 2 | Researchers must submit a cluster randomized trial involving human research participants for approval by a research ethics committee before commencing. | Some cluster randomized trials fail to report receiving research ethics approval. However, all research involving human participants must seek and obtain research ethics committee approval; this includes trials that evaluate minimal-risk quality improvement interventions (e.g. altering the hemodialysis central venous catheter care procedure compared to usual care). |
| Identifying research participants | 3 | Researchers should clearly identify the research participants in cluster randomized trials. A research participant can be identified as an individual whose interests may be affected as a result of study interventions or data collection procedures, that is, an individual:  (1) who is the intended recipient of an experimental (or control) intervention; or  (2) who is the direct target of an experimental (or control) manipulation of his/her environment; or  (3) with whom an investigator interacts for the purpose of collecting data about that individual; or  (4) about whom an investigator obtains identifiable private information for the purpose of collecting data about that individual.  Unless one or more of these criteria is met, an individual is not a research participant. | Most trials in hemodialysis setting include patients as research participants, while a few include health professionals as participants.  Health professionals are commonly overlooked as research participants; researchers and research ethics committees should pay special attention when a trial is testing interventions that specifically target these individuals. |
| Obtaining informed consent | 4 | Researchers must obtain informed consent from human research participants in a cluster randomized trial, unless a waiver of consent is granted by a research ethics committee under specific circumstances. | When informed consent is sought from participants, trials ought to report adequate details to assess the purpose of the consent (e.g., enrollment, receiving the intervention, data collection), from whom (e.g., patients, provider), when consent (before or after randomization), and how consent is obtained (e.g., written, oral). |
|  | 5 | When participants’ informed consent is required, but recruitment of participants is not possible *before* randomization of clusters, researchers must seek participants’ consent for trial enrollment as soon as possible after cluster randomization—that is, as soon as the potential participant has been identified, but before the participant has undergone any study interventions or data collection procedures. | Trials in hemodialysis setting often do no report the timing of informed consent or whether the consent procedures take place after randomization of clusters.  Obtaining consent post-randomization compromises randomization and increases the risk of recruitment bias. However, in hemodialysis trials, patients may need to be prospectively recruited after randomization (e.g., new patients starting hemodialysis treatment). Some authors put in place protections to reduce the risks of bias, including:  (1) ensuring that participants are recruited by individuals who are blinded to the cluster’s allocation;  (2) putting in place a standardized participant identification mechanism in all trial arms; and  (3) developing mechanisms whereby participants cannot become unblinded prior to consent or entry into the study. |
|  | 6 | A research ethics committee may approve a waiver or alteration of consent requirements when (1) the research is not feasible without a waiver or alteration of consent, and (2) the study interventions and data collection procedures pose no more than minimal risk.  Canada’s Tri-Council Policy Statement also require that: (3) the waiver or alteration is unlikely to adversely affect the welfare or the rights of participants, and (4) there is a plan to inform participants of the trial and the intervention, and participants can refuse the intervention | Hemodialysis trials utilizing waivers of consent commonly fail to adequately report how their study meets the criteria for a waiver or alteration of informed consent. |
|  | 7 | Researchers must obtain informed consent from professionals or other service providers who are research participants unless conditions for a waiver or alteration of consent are met. | While a few trials involve health care professionals as research participants, these individuals are commonly overlooked as research participants. Examples of health care providers who may be research participants in the hemodialysis setting include dialysis nurses, nurse educators, nurse practitioners, nephrologists, physiotherapists, kinesiologists, and pharmacists targeted by knowledge translation interventions. |
| Gatekeepers | 8 | Gatekeepers should not provide proxy consent on behalf of individuals in their cluster. | Gatekeepers in the hemodialysis setting may include but are not limited to medical directors, administrators, clinical care providers, members of patient and family advisory boards, members of the dialysis provider organization leadership, payers, or representatives of governmental organizations.  Gatekeepers can provide permission for the center to participate in the trial but cannot provide consent to study participation on behalf of patients who are research participants. |
|  | 9 | When a cluster randomized trial may substantially affect cluster or organizational interests, and a gatekeeper possesses the legitimate authority to make decisions on its behalf, the researcher should obtain the gatekeeper’s permission to enrol the cluster or organization in the trial. Such permission does not replace the need for the informed consent of research participants. | Trials in the hemodialysis setting rarely report the role of gatekeepers. Gatekeepers play an important role in hemodialysis trials as they usually provide permission for their cluster to be recruited or randomized to different arms of the trial. |
|  | 10 | When cluster randomized trial interventions may substantially affect cluster interests, researchers should seek to protect cluster interests through cluster consultation to inform study design, conduct, and reporting. Where relevant, gatekeepers can often facilitate such a consultation. | Hemodialysis trials rarely report whether any gatekeeper consultations take place. Researchers may consult with gatekeepers to facilitate implementation of the intervention, ensure high adherence to the assigned treatment protocol, and minimize or reduce protocol violations. |
| Assessing benefits and harms | 11 | The researcher must ensure that the study intervention is adequately justified. The benefits and harms of the study intervention must be consistent with competent practice in the field of study relevant to the cluster randomized trial. | Many aspects of clinical care in the hemodialysis setting are guided by clinical opinion and physiologic studies. There is a high degree of practice variations between hemodialysis centres and health care providers and this complicates the assessment of benefits and harms; which is often not reported in trials in the hemodialysis setting. |
|  | 12 | Researchers must adequately justify the choice of the control condition. When the control arm is usual practice or no treatment, individuals in the control arm must not be deprived of effective care or programmes to which they would have access, were there no trial. | Hemodialysis cluster randomized trials typically compare the effectiveness of existing, widely used interventions in the setting of routine clinical practice (i.e. usual care). Most trials utilize “usual care” as the control arm, however, there are instances where trials might conduct a head-to-head comparison between two interventions. |
|  | 13 | Researchers must ensure that data collection procedures are adequately justified. The risks of data collection procedures must (1) be minimised consistent with sound design and (2) stand in reasonable relation to the knowledge to be gained. | In the hemodialysis setting, trials commonly use routinely collected data (e.g. medical charts or electronic medical records) as a primary source for data collection and often supplement this information with other data sources (e.g. questionnaires, specimen collection, physical examination, administrative data, etc.). |
| Protecting vulnerable participants | 14 | Clusters may contain some vulnerable participants. In these circumstances, researchers and research ethics committees must consider whether additional protections are needed. | Kidney disease disproportionally affect individuals with multiple comorbidities, live in rural or remote locations, have dementia, lower education levels, and lower health literacy. These characteristics are also associated with poor comprehension of the informed consent processes.  Although vulnerable subgroups may have been included in the trials, none report putting protections in place for vulnerable populations. |
|  | 15 | When individual informed consent is required, and there are individuals who may be less able to choose participation freely because of their position in a cluster or organizational hierarchy, research ethics committees should pay special attention to recruitment, privacy, and consent procedures for those participants. | Researchers and research ethics committees must ensure the rights are protected for vulnerable participants (e.g. trainees or nurses) in an organizational setting, where a superior (e.g. medical director) might influence the participation in the study. However, without access to the original research ethics committee submission, it may not be possible to determine to what degree research ethics committees are attentive to recruitment, privacy, and consent procedures for such vulnerable participants. |

**Appendix 2:** PRISMA Checklist

| **Section/topic** | **#** | **Checklist item** | **Reported on page #** |
| --- | --- | --- | --- |
| **TITLE** | | |  |
| Title | 1 | Identify the report as a systematic review, meta-analysis, or both. | 1 |
| **ABSTRACT** | | |  |
| Structured summary | 2 | Provide a structured summary including, as applicable: background; objectives; data sources; study eligibility criteria, participants, and interventions; study appraisal and synthesis methods; results; limitations; conclusions and implications of key findings; systematic review registration number. | 2 |
| **INTRODUCTION** | | |  |
| Rationale | 3 | Describe the rationale for the review in the context of what is already known. | 3 / 4 |
| Objectives | 4 | Provide an explicit statement of questions being addressed with reference to participants, interventions, comparisons, outcomes, and study design (PICOS). | 4 |
| **METHODS** | | |  |
| Protocol and registration | 5 | Indicate if a review protocol exists, if and where it can be accessed (e.g., Web address), and, if available, provide registration information including registration number. | 4 |
| Eligibility criteria | 6 | Specify study characteristics (e.g., PICOS, length of follow-up) and report characteristics (e.g., years considered, language, publication status) used as criteria for eligibility, giving rationale. | 4 / 5 |
| Information sources | 7 | Describe all information sources (e.g., databases with dates of coverage, contact with study authors to identify additional studies) in the search and date last searched. | 5 |
| Search | 8 | Present full electronic search strategy for at least one database, including any limits used, such that it could be repeated. | 5 / Appendix 3 |
| Study selection | 9 | State the process for selecting studies (i.e., screening, eligibility, included in systematic review, and, if applicable, included in the meta-analysis). | 5 |
| Data collection process | 10 | Describe method of data extraction from reports (e.g., piloted forms, independently, in duplicate) and any processes for obtaining and confirming data from investigators. | 5 / 6 |
| Data items | 11 | List and define all variables for which data were sought (e.g., PICOS, funding sources) and any assumptions and simplifications made. | 5-6 / Appendix 4 |
| Risk of bias in individual studies | 12 | Describe methods used for assessing risk of bias of individual studies (including specification of whether this was done at the study or outcome level), and how this information is to be used in any data synthesis. | NA – 1 |
| Summary measures | 13 | State the principal summary measures (e.g., risk ratio, difference in means). | 6 |
| Synthesis of results | 14 | Describe the methods of handling data and combining results of studies, if done, including measures of consistency (e.g., I^2^) for each meta-analysis. | NA – 2 |
| Risk of bias across studies | 15 | Specify any assessment of risk of bias that may affect the cumulative evidence (e.g., publication bias, selective reporting within studies). | NA – 1 |
| Additional analyses | 16 | Describe methods of additional analyses (e.g., sensitivity or subgroup analyses, meta-regression), if done, indicating which were pre-specified. | NA – 2 |
| **RESULTS** | | |  |
| Study selection | 17 | Give numbers of studies screened, assessed for eligibility, and included in the review, with reasons for exclusions at each stage, ideally with a flow diagram. | 6 / Figure 1 |
| Study characteristics | 18 | For each study, present characteristics for which data were extracted (e.g., study size, PICOS, follow-up period) and provide the citations. | 6 / Table 1 |
| Risk of bias within studies | 19 | Present data on risk of bias of each study and, if available, any outcome level assessment (see item 12). | NA – 1 |
| Results of individual studies | 20 | For all outcomes considered (benefits or harms), present, for each study: (a) simple summary data for each intervention group (b) effect estimates and confidence intervals, ideally with a forest plot. | 7 to 10 |
| Synthesis of results | 21 | Present results of each meta-analysis done, including confidence intervals and measures of consistency. | NA – 2 |
| Risk of bias across studies | 22 | Present results of any assessment of risk of bias across studies (see Item 15). | NA – 1 |
| Additional analysis | 23 | Give results of additional analyses, if done (e.g., sensitivity or subgroup analyses, meta-regression [see Item 16]). | NA – 2 |
| **DISCUSSION** | | |  |
| Summary of evidence | 24 | Summarize the main findings including the strength of evidence for each main outcome; consider their relevance to key groups (e.g., healthcare providers, users, and policy makers). | 10 to 13 |
| Limitations | 25 | Discuss limitations at study and outcome level (e.g., risk of bias), and at review-level (e.g., incomplete retrieval of identified research, reporting bias). | 13 |
| Conclusions | 26 | Provide a general interpretation of the results in the context of other evidence, and implications for future research. | 14 |
| **FUNDING** | | |  |
| Funding | 27 | Describe sources of funding for the systematic review and other support (e.g., supply of data); role of funders for the systematic review. | 15 |

*From:*  Moher D, Liberati A, Tetzlaff J, Altman DG, The PRISMA Group (2009). Preferred Reporting Items for Systematic Reviews and Meta-Analyses: The PRISMA Statement. PLoS Med 6(7): e1000097. doi:10.1371/journal.pmed1000097

For more information, visit: **www.prisma-statement.org**.

NA – 1: The aim of this research was not to assess a specific health outcome within selected studies. But rather, we aimed to capture reporting of key ethical and methodological elements within selected hemodialysis cluster randomized trials.

NA – 2: While we do report some summary statistics (e.g. medians, range, etc.), the aim of this paper was not to capture measures of association for a specific outcome. Also, as described in the manuscript, given the small number of studies eligible for inclusion, we were unable to conduct any meaningful subgroup analyses.

**Appendix 3**: Search syntax to identify relevant articles in Medline between January 1^st^, 2000 and July 20^th^, 2018 in Embase Classic+Embase, Ovid MEDLINE(R) Epub Ahead of Print, In-Process & Other Non-Indexed Citations, Ovid MEDLINE(R) Daily and Ovid MEDLINE(R).

***Database: Ovid MEDLINE(R) ALL <1946 to November 30, 2019>***

-------------------------------------------------------------------------------

1. (dialy* or hemodi*).mp. or haemodi*.tw. or end-stage renal.tw. or endstage renal.tw. or end-stage kidney.tw. or esrd.tw. or renal replacement.mp. or uremia.mp. or uraemia.mp. or exp "Uremia"/ or capd.tw. or hemofilt*.mp. or haemofilt*.mp. or hyperphosphataemia.tw. or hyperphosphatemia.tw. or uremic patient*.tw. or uraemic patient*.tw. or secondary hyperparathyroidism.tw. or renal osteodystrophy.mp. or intradialy*.tw. or hyperoxaluria.mp. or tenckhoff*.tw. or autosomal dominant polycystic kidney.ti. or ccpd.tw. (241390)
2. exp Renal Insufficiency, Chronic/ (103332)
3. exp *kidney failure/ (113586)
4. (indwelling catheter/ or central venous catheterization/) and heparin.mp. (970)
5. ("Severity of Illness Index"/ or vascular.ti. or *"Anemia"/ or anemi*.ti. or anaemi*.ti. or nephrogenic.tw. or amyloid*.mp. or rhabdomyolysis.mp.) and *"Kidney Disease"/ (3849)
6. chronic.mp. and *"Kidney Disease"/ (11683)
7. ((kidney transplant* or renal transplant*) and (candidates or wait* list*)).tw. (2726)
8. encapsulating.tw. and scleros*.mp. (623)
9. or/1-8 (316044)
10. (random* and cluster*).mp. (31050)
11. (cluster* adj3 rct*).tw. (524)
12. (cluster* adj3 trial*).tw. (7928)
13. (communit* adj2 intervention*).tw. (6015)
14. (random* adj2 (group* or communit*)).tw. (20526)
15. or/10-14 (56430)
16. animals/ not humans/ (4443017)
17. 15 not 16 (51170)
18. 9 and 17 (448)
19. limit 18 to yr="2000 -Current" (383)
20. ("2017 06 23*" or "2017 06 24*" or "2017 06 25*" or "2017 06 26*" or "2017 06 27*" or "2017 06 28*" or "2017 06 29*" or "2017 06 3*" or 2017 07* or 2017 08* or 2017 09* or 2017 1* or 2018*).dt. (1375531)
21. 19 and 20 (32)

***Database: Embase Classic+Embase <1947 to November 30, 2019>***

-------------------------------------------------------------------------------

1. (dialy* or hemodi*).mp. or haemodi*.tw. or end-stage renal.tw. or endstage renal.tw. or end-stage kidney.tw. or esrd.tw. or renal replacement.mp. or uremia.mp. or uraemia.mp. or exp "Uremia"/ or capd.tw. or hemofilt*.mp. or haemofilt*.mp. or hyperphosphataemia.tw. or hyperphosphatemia.tw. or uremic patient*.tw. or uraemic patient*.tw. or secondary hyperparathyroidism.tw. or renal osteodystrophy.mp. or intradialy*.tw. or hyperoxaluria.mp. or tenckhoff*.tw. or autosomal dominant polycystic kidney.ti. or ccpd.tw. (357289)
2. *chronic kidney failure/ (39936)
3. *kidney failure/ or *end stage renal disease/ (45353)
4. (indwelling catheter/ or central venous catheterization/) and heparin.mp. (880)
5. ("Severity of Illness Index"/ or vascular.ti. or *"Anemia"/ or anemi*.ti. or anaemi*.ti. or nephrogenic.tw. or amyloid*.mp. or rhabdomyolysis.mp.) and (exp *kidney failure/ or *"Kidney Disease"/) (10012)
6. chronic.mp. and *"Kidney Disease"/ (9516)
7. ((kidney transplant* or renal transplant*) and (candidates or wait* list*)).tw. (5136)
8. encapsulating.tw. and scleros*.mp. (800)
9. or/1-8 (402695)
10. (cluster* and random*).tw. (34468)
11. (cluster* adj3 rct*).tw. (739)
12. (cluster* adj3 trial*).tw. (9762)
13. (communit* adj2 intervention*).tw. (7650)
14. (random* adj2 (group* or communit*)).tw. (30199)
15. controlled clinical trial/ and cluster analysis/ (952)
16. randomized controlled trial/ and cluster analysis/ (1870)
17. or/10-16 (71363)
18. (exp animal/ or nonhuman/) not exp human/ (6666585)
19. 17 not 18 (62060)
20. 9 and 19 (574)
21. limit 20 to yr="2000 -Current" (518)
22. ("20170623*" or "20170624*" or "20170625*" or "20170626*" or "20170627*" or "20170628*" or "20170629*" or "2017063*" or 2017 07* or 2017 08* or 2017 09* or 2017 1* or 2018*).dc. (1045427)
23. 21 and 22 (43)

**Appendix 4**: Extracted data

We extracted data on the following: **(1)** study characteristics, including year of publication, country of study recruitment, country’s level of development (for identification of emerging and developing economies, we used [World Economic outlook database](https://www.imf.org/external/pubs/ft/weo/2017/02/weodata/groups.htm)); **(2)** methodological characteristics, including study design, method of random allocation, unit of randomization, number of clusters and patients analyzed (or sample size estimated in the published protocol), data collection method, sample size estimation and whether clustering was taken into account, whether the analysis considered the effect of clustering; **(3)** justification for using a CRT design; **(4)** type of intervention and whom the intervention was targeting; **(5)** information regarding REC review, including which committee (e.g. local, central REC, etc.) reviewed the ethics application; **(6)** who provided access to the cluster and the role they played (i.e. any gatekeeper information). We defined a “Gatekeeper” as an individual or body that represents the interests of cluster members, clusters, or organizations.^1^ Gatekeepers may give permission to enroll the cluster in the trial, and when appropriate, give researchers the permission to approach eligible participants to enroll in the study**; (7)** information about informed consent procedures, including how (if at all) consent to the intervention and data collection was obtained, timing of participant consent, and what information (if any) was disclosed to participants during the consent procedure; and **(8)** any information about harm-benefit assessment or protection of vulnerable populations.

**Appendix 5**: Reported information about waiver of consent for the four studies that reported a waiver of informed patient consent or research ethic committee exemption.

| **Intervention and comparison groups** | **Outcome** | **Reported information about waiver of consent** |
| --- | --- | --- |
| Compared hemodialysis centres that used of Clear Guard HD Antimicrobial Barrier Caps versus hemodialysis centres that use of standard CVC caps | Positive blood culture rate as an indicator of blood stream infection rate | *“The informed consent waiver resulted in broad inclusion and ease of conducting the study.”*^2^ |
| Compared hemodialysis centres that used Clear Guard antimicrobial barrier caps with hemodialysis centres that used Tego hemodialysis connectors plus Curos disinfecting caps. | Blood culture positivity rate | *“The informed consent waiver was important for conducting the study in a pragmatic manner, adherence to the prescribed intervention, and broad inclusion”*^3^ |
| Compared hemodialysis that used 2% chlorhexidine with 70% alcohol swab sticks for exit-site care and 70% alcohol pads to perform “scrub the hubs” in dialysis-related central venous catheter care procedures with hemodialysis centres that used usual care. | Positive blood cultures for estimating blood stream infection rates. | *“…during its inception, because no investigational products were used and care processes in the intervention were all within standard clinical practice, this minimal-risk QI [Quality Improvement] initiative was not submitted for institutional review board review.”;*^4^ |
| Dialysis facilities randomized to the intervention adopted a default session duration of ≥4.25 hours (255 minutes) for patients initiating maintenance hemodialysis. If the treating nephrologist felt that the ≥4.25-hour duration was not appropriate for an individual patient, shorter treatments could be prescribed with the goal of achieving session durations as close to 4.25 hours as possible. Dialysis facilities randomized to usual care had no trial-driven approach to session duration. | Death | *"The trial was conducted under a waiver of the requirement for informed consent on the basis of criteria speciﬁed in the Common Rule [45 CFR Part 46.116(c)]. Patients in both intervention and usual care facilities were given written information about the trial that included the facility’s randomized assignment. Patients were provided with telephone access to the research teams at the dialysis provider organizations to obtain additional information and/or to opt out of having their clinical data included in the trial dataset. Patients meeting the eligibility criteria were enrolled in the trial unless they opted out of data sharing."*^5^ |

# References

1. Gallo A, Weijer C, White A, et al. What is the role and authority of gatekeepers in cluster randomized trials in health research? *Trials*. 2012;13(1):116. doi:10.1186/1745-6215-13-116

2. Hymes JL, Mooney A, Van Zandt C, Lynch L, Ziebol R, Killion D. Dialysis Catheter–Related Bloodstream Infections: A Cluster-Randomized Trial of the ClearGuard HD Antimicrobial Barrier Cap. *Am J Kidney Dis*. 2017;69(2):220-227. doi:10.1053/j.ajkd.2016.09.014

3. Brunelli SM, Van Wyck DB, Njord L, Ziebol RJ, Lynch LE, Killion DP. Cluster-Randomized Trial of Devices to Prevent Catheter-Related Bloodstream Infection. *J Am Soc Nephrol*. 2018;29(4):1336-1343. doi:10.1681/ASN.2017080870

4. Rosenblum A, Wang W, Ball LK, Latham C, Maddux FW, Lacson E. Hemodialysis catheter care strategies: A cluster-randomized quality improvement initiative. *Am J Kidney Dis*. 2014;63(2):259-267. doi:10.1053/j.ajkd.2013.08.019

5. Dember LM, Lacson E, Brunelli SM, et al. The TiME Trial: A Fully Embedded, Cluster-Randomized, Pragmatic Trial of Hemodialysis Session Duration. *J Am Soc Nephrol*. 2019;30(5):890-903. doi:10.1681/ASN.2018090945
